# Pennsylvania

**Published:** 1896-10

**Authors:** 


*Pennsylvania.* Tuberculosis has been discovered in a herd of cattle at Altoona. Some were tested, and one valuable Jersey cow condemned and destroyed by State Veterinarian Pearson.

A recent outbreak of disease among the cattle of Monroe County proved to be "red water," and not "anthrax." These animals had been grazing in forests which were burned over a year ago. The removal of the rest of the cattle from the woods ended the trouble. About sixty died before the nature of the disease was investigated.

